# Diagnostic ability of pancreatic juice cytology via the minor papilla in patients with pancreas divisum

**DOI:** 10.1002/deo2.62

**Published:** 2021-09-28

**Authors:** Shinya Kawaguchi, Tatsunori Satoh, Shuzo Terada, Shinya Endo, Naofumi Shirane

**Affiliations:** ^1^ Department of Gastroenterology Shizuoka General Hospital Shizuoka Japan

**Keywords:** minor papilla, pancreas divisum, pancreatic juice cytology, serial pancreatic juice aspiration cytologic examination

## Abstract

**Background and study aim:**

Endoscopic retrograde cholangiopancreatography (ERCP) is generally performed via the major papilla when evaluating patients with pancreatic disease. However, in patients with pancreas divisum (PD) or distortion of the main pancreatic duct, endoscopic retrograde pancreatography (ERP) should be performed via the minor papilla (MP). Our aim was to evaluate the efficacy and safety of endoscopic pancreatic juice cytology (PJC), performed via the MP, in patients with PD.

**Patients and methods:**

Patients with PD who underwent diagnostic ERP via the MP, between January 2010 and February 2021, were identified retrospectively from our hospital's ERCP database. Twenty‐two patients contributing to 24 ERCPs were included in the analysis.

**Results:**

MP cannulation was successful in 23 of 24 ERCPs (96%). In one patient, successful cannulation was achieved on the second attempt and the procedure was performed twice in another. Serial pancreatic juice aspiration cytologic examination (SPACE) was performed in 17 patients, with a single aspiration of pancreatic juice performed in the other five. The sensitivity, specificity, and accuracy rates of ERCP diagnosis, overall, were 56%, 100%, and 80%, respectively. When diagnosis only based on SPACE was considered, the accuracy rate was even higher at 87%. Three patients (13%) developed mild pancreatitis as an adverse event.

**Conclusions:**

The diagnostic ability of endoscopic PJC, via the MP in patients with PD was technically feasible and relatively effective under experienced pancreatobiliary endoscopists, however, requiring careful attention to post‐ERCP pancreatitis when performed.

## INTRODUCTION

Endoscopic retrograde cholangiopancreatography (ERCP) is generally performed via the major papilla when evaluating patients with pancreatic disease. However, for patients with pancreas divisum (PD) or distortion of the main pancreatic duct (MPD), endoscopic retrograde pancreatography (ERP) should be performed via the minor papilla (MP).

The incidence rate of PD is 5%–10% in Western countries and only 0.6%–1.2% in Japan.^1^, ^2^, ^3^ The incidence rate of complications for PD and pancreatic cancer is estimated at 5.1%–12.2% in Europe and the United States, but with no consensus on the relationship between pancreatic cancer and PD.^4^, ^5^, ^6^ Traverso et al.^7^ speculated that long‐standing dorsal duct obstruction due to MP stenosis may be a promoting factor of PD and pancreatic cancer. In cases of PD, extensive epithelial hyperplasia, with atypia, high‐grade pancreatic intraepithelial neoplasia (PanIN), and focal invasive adenocarcinoma have been observed along the dilated dorsal pancreatic duct, with normal epithelium lining the ventral duct. As patients with PD may be at high risk for pancreatic malignant diseases, we have been actively performing pancreatic juice cytology (PJC), via the MP, at our hospital. The diagnostic accuracy rate for PJC through a nasopancreatic drainage (NPD) catheter, known as serial pancreatic juice aspiration cytologic examination (SPACE) is reported to be 87%, which is higher than the accuracy rate for conventional diagnostics of early‐stage pancreatic cancer.^8^ However, in some patients with PD, MP cannulation is difficult due to uncertainty in identifying the papilla or the absence of a visible opening.^9^ Moreover, it may be difficult to inject a contrast medium into the MPD owing to the small size of the orifice of the MP compared with that of the major papilla.^10^ To date, however, there have been no comprehensive reports regarding the use of PJC via the MP in patients with PD. Our study addresses this gap in the literature, with the aim of evaluating the efficacy and safety of the endoscopic PJC, via the MP, in patients with PD.

## PATIENTS AND METHODS

### Study population

**FIGURE 1:**
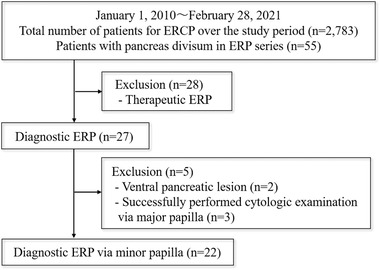
Patient eligibility flowchart

The ERCP database at our hospital, Shizuoka General Hospital, was retrospectively examined to identify patients with PD who had undergone diagnostic ERP via the MP between January 2010 and February 2021 (Figure 1).

### Clinical outcomes

The following details were extracted from patient records for analysis: the patient's clinical profile, outcomes of endoscopic interventions via the MP, and complications associated with these endoscopic procedures. Incomplete PD is a pancreatic anomaly characterized by inadequate communication between the ventral and dorsal pancreatic duct and is classified into three subtypes.^11^, ^12^ Post‐ERCP pancreatitis (PEP), a major complication of endoscopic interventions, was diagnosed based on the criteria proposed by Cotton et al.^13^ as pancreatic pain and hyperamylasemia occurring within 24 h of the procedure. Pancreatic pain was defined as persistent pain in the epigastric or periumbilical regions and hyperamylasemia as an increase in the serum amylase level to more than three times the upper normal limit. The lexicon for endoscopic adverse events (AEs), advocated by the American Society of Gastrointestinal Endoscopy, was used to diagnose and grade the severity of the other AEs.^14^

### ERCP devices and MP cannulation

**FIGURE 2:**
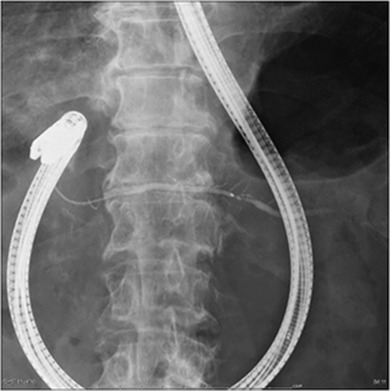
Fluoroscopic images showing that the side‐viewing duodenoscope to be set to the push position for endoscopic retrograde pancreatography (ERP) via the minor papilla (MP)

**FIGURE 3:**
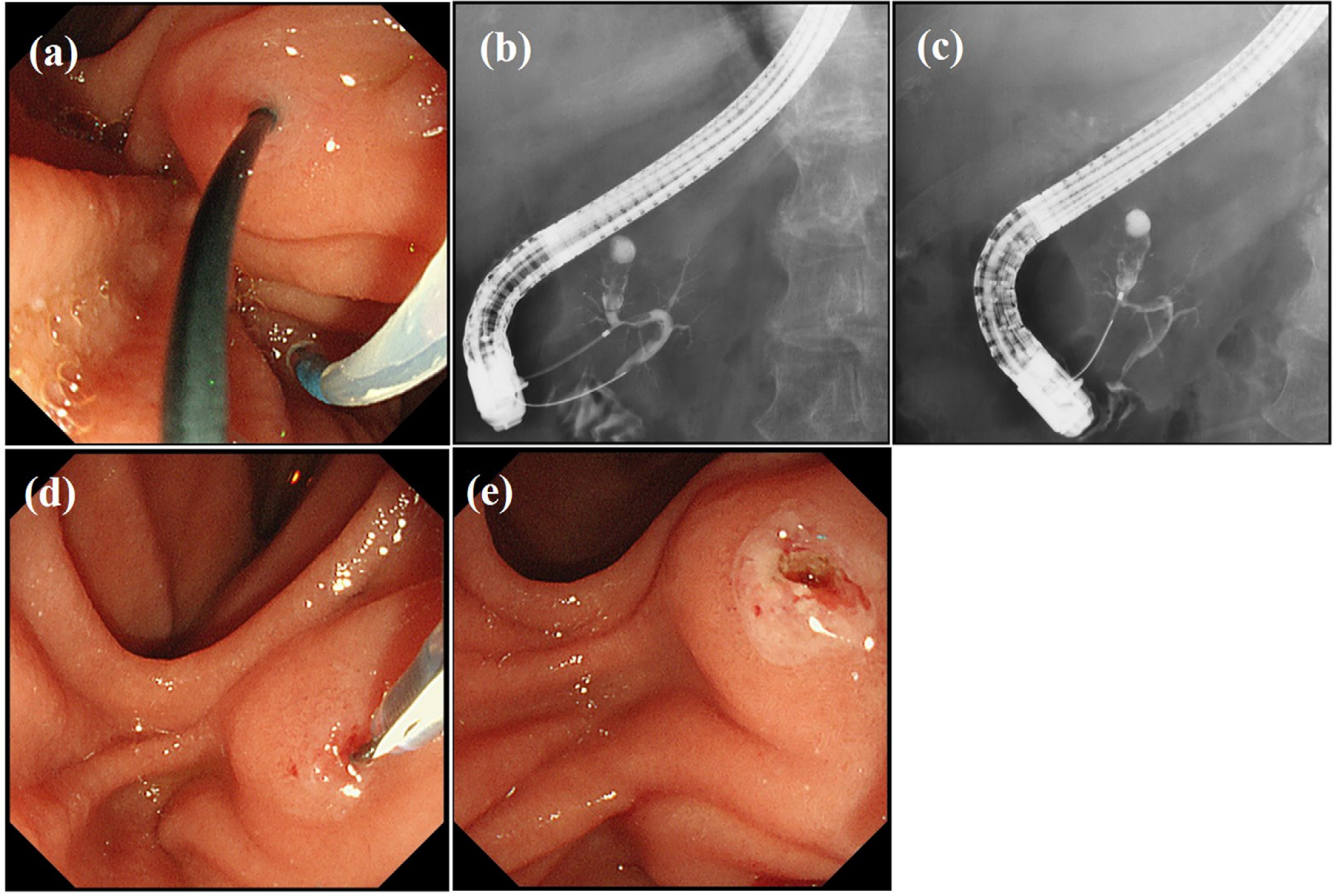
Endoscopic and fluoroscopic images showing insertion of a guidewire from the major papilla into the duodenum through the duct of Santorini and the minor papilla (MP) (a,b). Fluoroscopic image showing the insertion of a catheter via the MP (rendezvous technique) (c). Endoscopic image showing endoscopic MP sphincterotomy performed using a needle‐knife (d,e)

A side‐viewing duodenoscope (JF‐260V, TJF‐260, and TJF‐290V; Olympus Medical Systems, Tokyo, Japan) was used for all ERCP procedures, with the major first cannulated using a standard catheter (ERCP catheter; MTW Co. Ltd, Dusseldorf, Germany). When endoscopists judged access to the MPD via the major papilla difficult due to PD, cannulation of the MPD was attempted. For ERP via the MP, the side‐viewing duodenoscope was set to the push position (Figure 2). The MP was usually cannulated using a tapered catheter (PR‐110Q; Olympus Medical Systems), with a guidewire (Jagwire; 0.025 inch in diameter, Boston Scientific, Tokyo, Japan and M‐Through; 0.025 inch in diameter, Asahi Intecc, Aichi, Japan). When contrast medium could not be injected, wire‐guided cannulation (WGC) was attempted. When a direct approach through the MP was still difficult, insertion of a guidewire through the major papilla was attempted. After insertion of a guidewire into the duct of Wirsung, the guidewire was pushed into the duodenum through the duct of Santorini and the MP where it was grasped and removed using the biopsy channel of the duodenoscope (rendezvous technique)^15^ (Figure 3a–c). When deep cannulation was difficult because the MP was small, endoscopic MP sphincterotomy was performed using a needle‐knife (KD‐10Q‐1; Olympus Medical Systems) positioned adjacent to a previously inserted guidewire, taking care not to cut too much. (Figure 3d,e). A secondary three‐radial star‐shaped papilla incision was made with a needle‐knife. All examinations were performed under the supervision of two pancreatobiliary endoscopists who had experienced performing >400 ERCP procedures annually.

### NPD catheter placement and SPACE or PJC

We used a 5‐Fr pigtail NPD catheter (QuickPlaceV; Olympus) to collect up to six samples of pancreatic juice per session for 2 days, with the NPD catheter subsequently removed.^16^ For cases with pancreatic duct stenosis, the tip of the NPD catheter was placed across the stenosis. We do note that some patients underwent a single aspiration of pancreatic juice, at the discretion of the endoscopist. The indications for a single aspiration of pancreatic juice and SPACE have changed over the time period of our study. At present, we consistently use SPACE for NPD placement. Positive results included a diagnosis of adenocarcinoma or suspicious adenocarcinoma.

### Diagnostic accuracy rate of PJC

The final diagnosis of malignancy was comprehensively judged based on the surgical pathological findings or clinical course over a period of 6 months after the examination. A benign diagnosis was based on the absence of abnormal surgical pathological findings or no change in imaging findings for a duration >2 years after the examination.

## RESULTS

### Clinical profiles of the study group

A total of 5603 ERCPs were performed in 2783 patients, including ERCP performed in 683 patients for ERP purposes over the study period. Of these 683 patients, 55 were diagnosed with PD based on the ERP findings. Of these 55 patients, 28 patients were excluded as they had received therapeutic procedures for chronic pancreatitis or pancreatic lesions. Of the remaining 27 patients, cytologic examination via the major papilla was performed in two patients with ventral pancreatic lesions and three patients with dorsal pancreatic lesions. The remaining 22 patients underwent diagnostic ERP via the MP and were included in our analysis (Figure 1).

Patient demographics and indications for ERP procedure are summarized in Table 1. The list of patients is shown in Tables S1 and S2. The study group of 22 patients included 13 men, with a median age of 73 (range, 43–89) years, of whom nine had a diagnosis of complete PD and 13 of incomplete PD. With regard to the subtypes of incomplete PD, three patients had subtype 2 and 10 had subtype 3. Endoscopic ultrasound (EUS) was performed in all patients before ERP, with findings of EUS used to identify the possibility of malignancy for which ERP for PJC is indicated. The most common indication for diagnostic ERP was cystic neoplasm, such as intraductal papillary mucinous neoplasm (IPMN) with high‐risk stigmata (HS) or worrisome features (WF), in 12 patients. The definition of HS or WF was as per the revised definitions of the 2017 International Consensus Fukuoka guidelines.^17^ Other indications for diagnostic ERP were pancreatitis with suspected tumor‐induced changes in the MPD in three patients, presence of a small pancreatic mass, approximately 10 mm in diameter that was deemed difficult to diagnose by EUS‐fine needle aspiration (FNA) in three patients, focal MPD stricture in three patients, and MPD dilatation in one patient.

**TABLE 1:** Patient demographics and indications for the procedure

Number of patients	22
Male (*N*)	13
Median age (range) (years)	73 (43–89)
ERP sessions via minor papilla (*N*)	24
Total number of patients for ERCP during the study period	2783
Total number of patients for ERP during the study period	683
Type of PD	
Complete (*N*)	9
Incomplete (subtype 2) (*N*)	3
Incomplete (subtype 3) (*N*)	10
Indications for diagnostic ERP	
Cystic neoplasm (*N*)	12
IPMN with high‐risk stigmata (*N*)	5
IPMN with worrisome feature (*N*)	7
Pancreatitis (*N*)	3
Small pancreatic mass, approximately 10 mm in diameter (*N*)	3
Focal MPD stricture (*N*)	3
MPD dilatation (*N*)	1

Abbreviations: IPMN, intraductal papillary mucinous neoplasm; ERCP, endoscopic retrograde cholangiopancreatography; ERP, endoscopic retrograde pancreatography; MPD, main pancreatic duct; PD, pancreas divisum.

### MP cannulation and NPD catheter placement

**FIGURE 4:**
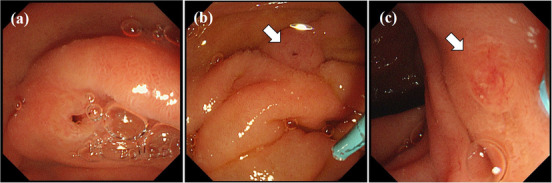
Endoscopic image of the orifice of the minor papilla (MP) during the cannulation showing dilatation (a), mild dilation (b) (arrow), and without dilation (c) (arrow)

**FIGURE 5:**
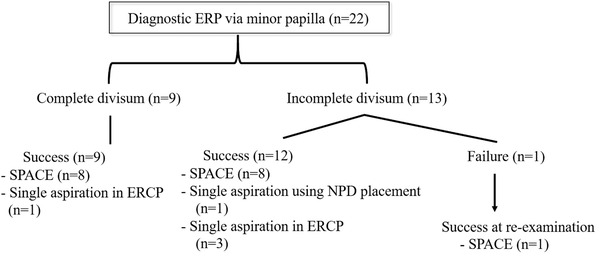
Outcomes of the diagnostic endoscopic retrograde pancreatography (ERP) and nasopancreatic drainage (NPD) placement via minor papilla (MP)

The 22 patients included in the analysis contributed 24 ERCPs. MRCP was performed in 19 patients, with PD diagnosed in eight of these patients. Contrast injection via the major papilla for the purpose of confirming PD and was useful for ERP strategy. MP cannulation was successful in 23 of 24 (96%) ERCPs, with successful MP cannulation achieved on the second attempt for the remaining patient. Of note, ERP and SPACE were repeated twice in one patient. During cannulation via the MP, dilation of the orifice of the MP was observed in six of the nine patients (67%) with complete PD and in three of the 13 patients (23%) with incomplete PD (Figure 4). For cannulation, contrast injection technique, using a tapered tip catheter with or without guidewire, was used in 13 patients (14 ERPs), a WGC in five, a rendezvous technique in four, and a precut incision in one patient (Table 2). Among these nine cases of complete PD in our study, cannulation via the MP was initially started in three cases. The median procedure time for ERP via the MP was 30 (range, 8–66) min, increasing to 42 (range, 23–66) min for these four cases in whom the rendezvous technique was used. The NPD catheter was successfully placed, via the MP, in 18 patients. SPACE was discontinued in one patient due to spontaneous dislodging of the catheter. SPACE was successfully performed in 17 patients. A single aspiration of pancreatic juice was obtained during the ERP procedure in five patients (Figure 5).

**TABLE 2:** Minor papilla (MP) cannulation and nasopancreatic drainage (NPD) catheter placement (*N*)

Contrast injection technique	14
Wire‐guided cannulation	5
Rendezvous technique	4
Precut method	1
Additional precut before NPD catheter placement	5

### Sensitivity, specificity, and accuracy rate of PJC

Two patients with a follow‐up period of <2 years were excluded from the analysis. Pathological examination of the surgical specimen was performed in eight of nine cases with malignant findings and two of 11 with benign findings. The malignancy rate for the remaining cases without surgical specimens was judged based on the clinical course over a period of 6 months and 2 years after the examination for cases with malignant and benign findings, respectively. The follow‐up period for the 22 patients in the analysis ranged from 45 months to 128 months. SPACE was performed in 17 patients, with a single aspiration of pancreatic juice obtained during the ERCP procedure in the other five patients. The sensitivity, specificity, and accuracy rate of PJC were 56% (5/9 patients), 100% (11/11 patients), and 80% (16/20 patients), respectively. When only considering the results for SPACE, these results were even higher at 67% (4/6 patients), 100% (9/9 patients), and 87% (13/15 patients), respectively (Table 3). The final diagnosis was invasive pancreatic ductal adenocarcinoma in three patients, high‐grade PanIN without invasive carcinoma in two, intraductal papillary mucinous carcinoma in four, intraductal papillary mucinous adenoma in two, benign IPMNs (based on follow‐up) in four, idiopathic pancreatitis in two, tumor‐forming pancreatitis in one, benign MPD stricture in one, and benign MPD dilatation due to incomplete PD in one patient. Tumor staging, based on the Union for International Cancer Control (UICC) (8th edition), for patients with malignancy diagnosed at a relatively early stage, was as follows: Stage 0, three patients; stage I, four patients; stage III, one patient; and stage IV (para‐aortic lymph node metastasis), one patient.

**TABLE 3:** Diagnostic ability of pancreatic juice cytology

Malignancy (*N*)	9	SPACE	
High‐grade PanIN (*N*)	2	Sensitivity	67% (4/6)
Invasive PDAC (*N*)	3	Specificity	100% (9/9)
IPMC (*N*)	4	Accuracy rate	87% (13/15)
Stage 0/I/III/IV (*N*)	3/4/1/1	Single aspiration of pancreatic juice	
		Sensitivity	33% (1/3)
		Specificity	100% (2/2)
		Accuracy rate	60% (3/5)
		Total	
		Sensitivity	56% (5/9)
		Specificity	100% (11/11)
		Accuracy rate	80% (16/20)

Abbreviations: IPMC, intraductal papillary mucinous carcinoma; PanIN, pancreatic intraepithelial neoplasm; PDAC, pancreatic ductal adenocarcinoma; SPACE, serial pancreatic juice aspiration cytologic examination; Stage, UICC (8th edition).

### Adverse events associated with ERCP

There were no AEs, such as bleeding or perforation, associated with endoscopic MP sphincterotomy. However, three patients (13%) developed mild pancreatitis, but PEP resolved promptly with conservative treatment in all three patients. These three cases were diagnosed with complete PD in the first case (Case 1) and incomplete PD in the second and third cases (Cases 12 and 13), the details of which are described below. In the first case, contrast injection was performed for IPMN with WF, which required 16 min to be performed and resulted in dorsal pancreatitis. In the second case, a rendezvous technique and precut method were performed for pancreatic ductal dilatation, which required 42 min and resulted in ventral pancreatitis. In the third case, contrast injection was performed for IPMN with WF, which required 28 min to performed and resulted in ventral pancreatitis.

## DISCUSSION

Of the 683 ERPs performed in 2783 patients, PD was diagnosed in 55 (8.1%), based on the ERP findings. This frequency of PD is higher than in previous reports published in Japan, likely because we limited our study to patients with pancreatic diseases. Our MP cannulation success rate was 96% (23/24 ERCPs). The sensitivity, specificity, and accuracy rate of SPACE were 67%, 100%, and 87%, respectively. The complication rate was 13%, with three of 22 patients developing mild pancreatitis.

Endoscopic diagnosis of pancreatic diseases is usually performed via the major papilla. However, the major papilla is sometimes difficult to approach in patients with PD. In these difficult cases, an approach via the MP is attempted as the only alternative, although MP cannulation remains challenging in these cases, even for experienced endoscopists. The European Society of Gastrointestinal Endoscopy suggests using WGC, with or without contrast, and sphincterotomy, using a pull‐type sphincterotome or a needle‐knife over a plastic stent.^9^ When cannulation of the MP is difficult, the European Society of Gastrointestinal Endoscopy suggests using secretin injection, which can be preceded by the use of methylene blue spray in the duodenum.^9^ A success rate of cannulation via the MP of 74%–86% has been reported in patients with recurrent acute pancreatitis with PD.^18^, ^19^, ^20^ To our knowledge, however, our study is the first comprehensive report on the success rate of cannulation, via the MP, and PJC for diagnostic purposes in patients with PD. Based on our experience, we believe that insertion of the guidewire into the duct of Santorini is the most important step during the procedure, however, this does require close cooperation between the endoscopist manipulating the catheter and the assistant advancing the guidewire to prevent PEP.^10^ In most cases, we chose to perform ERP via the major papilla first based on the following reasons. The first is the pooled sensitivity and specificity of MRCP for diagnosis of PD of 52%,^21^ of which the second being the ERCP is generally performed using cannulation via the major papilla, with this technique being well‐established and stable. In cases of complete PD, such as cases of IPMN with MP dilatation, cannulation via the MP should be the first option in our view. In our study sample, although pancreatography was possible, deep cannulation was difficult because the MP was small. A precut MP sphincterotomy was performed with a needle‐knife beside a previously inserted guidewire, taking care not to cut too much. Following this precut, a 5‐Fr pigtail NPD catheter could be successfully placed.

The diagnostic accuracy rate of 87 % using PJC via an NPD catheter is higher than the accuracy rate for the conventional method.^8^ This may reflect the fact that high‐grade PanIN does not always occur from the MPD but may, in fact, also occur from branches of the pancreatic duct.^8^, ^14^, ^22^ In these cases, SPACE would be more sensitive than ordinary cytologic examination, such as brushing cytology.^8^ In Japan, ERCP is often selected for endoscopic PJC in patients with suspected IPMN. According to the most widely used revision of the International Consensus Fukuoka guidelines, namely the 2017 guidelines, for management of IPMN of the pancreas, it is important to highlight that Japanese investigators do not recommend EUS‐guided fine needle aspiration for the diagnosis of mucinous‐like cystic lesions with HS or WF as it may cause leakage of the cyst content, potentially leading to peritoneal dissemination or gastric seeding.^23^, ^24^ Therefore, SPACE has an important role in diagnostic evaluation in Japan.

In their case series of 26 patients who underwent diagnostic ERP, Fujimori et al. reported successful cannulation of the MP in 19 patients. Of these, eight underwent diagnostic pancreatography only and 11 underwent PJC, including four patients in whom SPACE was used for the evaluation of MPD‐type IPMN.^25^ In our study, MP cannulation was successful in 23 of 24 ERCP procedures, with the successful placement of the NPD catheter via the MP in 18 patients. However, SPACE was discontinued in one of these 18 cases due to spontaneous dislodging of the catheter, with successful SPACE performed in the other 17 patients. The accuracy rate of SPACE was 87%, which was comparable to a previously reported rate.^8^

There were no AEs associated with endoscopic MP sphincterotomy, such as bleeding or perforation. Three patients did develop mild pancreatitis, with prompt resolution of PEP achieved in all patients with conservative treatment. Moffatt et al. reported that patients with PD who undergo MP cannulation, with or without MP sphincterotomy, are at high risk for PEP (10.2% with and 8.2% without MP sphincterotomy).^26^ Therefore, endoscopic MP intervention is regarded as more hazardous than typical ERCP techniques.^27^

The limitations of our study need to be acknowledged in the interpretation of our results. Foremost, is the retrospective and single‐center design, with small sample size. As such, optimal indications for PJC via the MP are unclear. Multicenter studies with larger sample sizes are required to fully assess the efficacy and safety of this procedure.

In conclusion, the diagnostic ability of PJC via the MP in patients with PD was technically feasible and effective under experienced pancreatobiliary endoscopists, however, requiring careful attention to PEP when performed.

## ETHICS STATEMENT

All patients provided written informed consent for ERCP, including endoscopic procedures. Our institutional review board (SGHIRB#2020058) approved our retrospective study, with the need for informed consent waived. Our study was performed in accordance with the principles of the Declaration of Helsinki (current revision, October 2013).

## CONFLICT OF INTEREST

The authors declare that they have no conflict of interest.

## FUNDING INFORMATION

None.

## Supporting information


**Table S1** Indication for diagnostic ERP and diagnoses
**Table S2** Procedures for MP cannulation and AEs
